# Urinary Hippuric Acid as a Sex-Dependent Biomarker for Fruit and Nut Intake Raised from the EAT-Lancet Index and Nuclear Magnetic Resonance Analysis

**DOI:** 10.3390/metabo15060348

**Published:** 2025-05-23

**Authors:** Edwin Fernández-Cruz, Víctor de la O, Cristina M. Fernández-Diaz, Pilar Matía-Martín, M. Ángel Rubio-Herrera, Nuria Amigó, Alfonso L. Calle-Pascual, J. Alfredo Martínez

**Affiliations:** 1 IMDEA Food, CEI UAM + CSIC, Carretera de Cantoblanco 8, 28049 Madrid, Spain; victor.delao@unir.net (V.d.l.O.); cristinamaria.fernandez@imdea.org (C.M.F.-D.); 2 Faculty of Health Sciences, International University of La Rioja (UNIR), 26006 Logroño, Spain; 3 Endocrinology and Nutrition Department, San Carlos Clinical Hospital and Instituto de Investigación Sanitaria San Carlos (IdISSC), 28040 Madrid, Spain; mariapilar.matia@salud.madrid.org (P.M.-M.); miguelangel.rubio@salud.madrid.org (M.Á.R.-H.); 4 Centro de Investigación Biomédica en Red de Diabetes y Enfermedades Metabólicas Asociadas (CIBERDEM), 28029 Madrid, Spain; alfonsoluis.calle@salud.madrid.org; 5 Department of Medicine II, Faculty of Medicine, Complutense University of Madrid, 28040 Madrid, Spain; 6 Departament de Ciències Mèdiques Bàsiques, Universitat Rovira i Virgili, 43201 Tarragona, Spain; nuria.amigo@urv.cat; 7 Centro de Investigación Biomédica en Red Fisiopatología de La Obesidad y La Nutrición (CIBEROBN), Institute of Health Carlos III, 28029 Madrid, Spain; 8 Department of Medicine and Endocrinology, Campus of Soria, University of Valladolid, 42004 Soria, Spain

**Keywords:** biomarker of food intake, dietary assessment, EAT-Lancet index, hippuric acid, precision nutrition

## Abstract

**Background/Objectives**: Assessing nutrient intake is essential for understanding body homeostasis and diet–health interactions. Traditional methods, such as dietary questionnaires and quality indices, are limited by subjectivity and variability in food composition tables. Metabolomic markers, like urinary hippuric acid, provide an objective means to estimate food and nutrient intake, helping to link dietary patterns with metabolic outputs and health outcomes. This study uniquely evaluates urinary hippuric acid as a putative biomarker of nut intake, expanding the previously known role as a fruit intake marker, and investigates sex-related differences in the excretion. **Methods**: Using Nuclear Magnetic Resonance (NMR) spectroscopy, 34 urinary metabolites from 138 participants (69.7% women) in the Dietary Deal project were analyzed. Metabolite concentrations were categorized by median adherence to the EAT-Lancet score (≤p50 or >p50). A validated Food Frequency Questionnaire (FFQ) assessed dietary and energy intake. Correlation analyses linked metabolites to the 14 EAT-Lancet food groups, and a linear regression adjusted model examined associations between urinary hippuric acid and fruit/nut consumption, with sensitivity analysis for sex. **Results**: The EAT-Lancet index, stratified by median adherence, effectively distinguished between high and low dietary intake of fruits (*p* = 0.012) and nuts (*p* < 0.001). Urinary hippuric acid concentrations were found to be influenced by sex (*p* = 0.020), with females showing a 44.7% higher mean concentration. Overall, urinary hippuric acid levels were positively associated with FFQ-estimated nut consumption (*p* = 0.049), providing the first evidence of potential suitability as a nut intake biomarker. **Conclusions**: Hippuric acid emerges as a promising dietary biomarker for assessing nut intake in healthy populations. This study provides novel insights that extend the application of hippuric acid to dietary nut assessment and emphasizes the importance of a sex-specific interpretation for precision nutrition purposes using NMR technology.

## 1. Introduction

Nutrition plays a critical role in maintaining health, preventing chronic diseases, and contributing to effective diet-based therapeutic strategies [1]. However, unhealthy dietary habits are major contributors to the global burden of chronic diseases, including obesity, cardiovascular events, type 2 diabetes, and certain cancers [2]. In this context, improving our understanding of the influence of nutrition on health is essential for developing targeted and evidence-based dietary interventions and public health policies.

Accurately assessing food and nutrient intake remains a key aspect of personalized nutritional advice and public health strategies [3]. However, traditional dietary assessment methods such as 24 h recalls, food frequency questionnaires (FFQ), and dietary records, are based on self-reported information, which is susceptible to various sources of bias, including underreporting, recall errors, and portion size estimation difficulties, in addition to food composition inaccuracies in existing databases [4,5]. To address these limitations, diet quality indices like the Alternate Healthy Eating Index (AHEI), the Dietary Approaches to Stop Hypertension (DASH) score, and the 14-item Mediterranean Diet Adherence Screener (14-MEDAS) have been used to summarize overall diet quality [6,7]. Recently, the EAT-Lancet score has emerged as a widely accepted index for evaluating adherence to dietary patterns with implications for both human and global health [8,9].

Despite dietary quality indices utility, these methods still lack objectivity and validation, since they rely on indirect indicators of food intake [5]. Consequently, researchers and nutritionists have shown a growing interest in dietary biomarkers—measurable biological compounds found in biofluids such as urine or blood—that can reflect actual food or nutrient intake more accurately than traditional self-reported methods [10,11]. Metabolomics, especially using Nuclear Magnetic Resonance (NMR) spectroscopy, offers an untargeted approach to identify metabolic fingerprints associated with dietary patterns and specific food intake [12,13]. For instance, proline betaine is associated with citrus fruits [14], trimethylamine-N-oxide with fish consumption [15], 3-methylhistidine with meat intake [16], and alkylresorcinol metabolites with grain consumption [17], among others, where advanced methodologies can facilitate the metabolomic approach. Also, NMR methodology has enabled the detection of a wide variety of metabolites derived from specific dietary components, particularly those abundant in plant-based foods, with an affordable methodology. Among them, polyphenols—naturally occurring compounds with antioxidant properties—have drawn significant attention due to their wide distribution in the diet and their impact on human health [18]. Dietary polyphenols are abundant in a wide range of foods, mainly fruits, vegetables, and beverages such as fruit juices, tea, coffee, and wine, and are extensively metabolized by the gut microbiota into smaller phenolic acids [19]. However, spices, herbs, nuts, seeds, and olive oil are also notable dietary sources of these compounds [20].

One of the most studied final products of microbial metabolism is hippuric acid, a conjugate of benzoic acid and glycine that is ultimately excreted in urine [21]. As such, urinary hippuric acid has consistently emerged as a potential biomarker of polyphenol-rich food intake, especially fruits and vegetables [22]. Nevertheless, the potential of this compound as a biomarker of other foods, such as nuts, has been scarcely explored and remains unvalidated in adult populations. Although some metabolomic studies have suggested that nuts may contribute to hippuric acid production due to their ellagitannin and proanthocyanidin content, consistent evidence is still lacking [23,24]. In this context, NMR offers a unique value for assessing dietary intake through urinary metabolites in comparison to High-Performance Liquid Chromatography (HPLC) and Gas Chromatography (GC) technologies [25].

Furthermore, biological sex influences nutrient metabolism, gut microbiota composition, and metabolic responses to dietary intake [26,27]. Sex-specific differences have been reported in polyphenol absorption, biotransformation, and excretion, which may affect the interpretation of dietary biomarkers such as hippuric acid [28]. Despite growing interest in personalized nutrition, few studies have simultaneously assessed dietary intake, urinary metabolomic profiles, and sex-specific differences in free-living populations [29]. This issue represents a relevant gap in the field, particularly in the context of precision nutrition, which aims to tailor dietary recommendations based on individual biological characteristics.

The aim of the present study is to evaluate the association between adherence to dietary pattern (as measured by the EAT-Lancet score) and urinary hippuric acid concentrations, while exploring sex-specific differences in this association. We hypothesized that urinary hippuric acid may serve as a biomarker of nut intake and that this relationship may be influenced by sex. By investigating hippuric acid as a sex-dependent biomarker of nut consumption, this study may contribute novel insights into the future validation of dietary biomarkers and support the integration of metabolomics into personalized nutrition strategies.

## 2. Materials and Methods

### 2.1. Study Design and Population

An ancillary observational pilot study was conducted as part of the Dietary Deal project (ref. AC21/00038). Approval for the clinical trial was granted by the Ethics Committee of the Hospital Clínico San Carlos on 7 June 2022 (Code 22/363-E). A total of 138 adult participants of both sexes were recruited from the Endocrinology Department at Hospital Clínico San Carlos (Madrid, Spain) between November 2022 and June 2023. The inclusion criteria required male and female volunteers aged between 18 and 75 years, with a medical record number at the hospital, an adequate educational level and understanding of the nutritional study, and a willingness to voluntarily participate by providing written informed consent. Exclusion criteria included dementia, mental illness, cognitive impairment, participation in other parallel clinical trials, or the use of pharmacological treatments that could alter metabolite levels. Participation required compliance with all the inclusion criteria. This study included a single initial visit, where data on dietary intake, body composition, anthropometric measurements, quality of life, physical activity, and biochemical variables were collected for analysis and deposition.

Recruitment was carried out through medical specialists and staff from the Endocrinology Department at Hospital Clínico San Carlos. Patients attending outpatient consultations were informed about the study and provided with an information sheet and informed consent form, after which a date was scheduled for the study visit. Once enrolled, each participant was required to attend a single hospital visit, arriving after at least 12 h of fasting and bringing a 3-day dietary recall covering the days prior to the visit. Urine samples were collected from the first morning void and stored in sterile 150 mL containers, from which four 2 mL aliquots were extracted for subsequent analysis, following validated criteria. All aliquots were stored at −80 °C in an auto-monitored freezer until sample analysis. Subsequently, anthropometric measurements were taken, including body weight and height using a Seca 220 stadiometer, and body composition using a Seca mBCA 515^®^ device, which was also used to calculate Body Mass Index (BMI), calculated as [weight (kg)/height^2^ (m^2^)] as previously described [30].

Later, a registered dietitian/nutritionist collected the signed informed consent forms, reviewed the 72 h dietary recalls, and administered a validated 136-item FFQ [31]. Then, the International Physical Activity Questionnaire Short Form (IPAQ-SF) was used to evaluate physical activity through the frequency and duration of moderate and vigorous activities, walking, and sedentary time over the past week, as detailed elsewhere [32]. The EAT-Lancet diet score was calculated following the methodology described by the EPIC-Oxford study [8]. The score is based on 14 dietary components aligned with the EAT-Lancet Commission’s reference diet. One point was assigned for each component if the intake met the predefined recommendation, resulting in a total score ranging from 0 to 14. Individual dietary intakes were estimated using the validated 136-item FFQ, and the EAT-Lancet score was subsequently derived from the FFQ responses.

### 2.2. ^1^H-NMR Urine Metabolomics

Urine samples were prepared and analyzed using standardized and optimized protocols as previously described [33]. Briefly, urine samples were thawed at room temperature (minimum 1 h) and homogenized by gently inverting the tubes twice before 540 μL of urine was mixed with 60 μL of buffer (KH_2_PO_4_, 1.5 M, pH 7.4 made up in 2H_2_O) containing 5.8 mM TSP and 2 mM NaN_3_. Following centrifugation (1800× *g*, 4 °C, 5 min) to remove solids, 550 μL of the sample was transferred into 5 mm SampleJet NMR tubes and immediately loaded onto a refrigerated SampleJet robot (Bruker Biospin, Rheinstetten, Germany) and maintained at 4 °C until NMR analysis. Before acquisition, the samples were allowed to equilibrate inside the spectrometer for at least 5 min to ensure thermal stability. Acquisition routines, including temperature equilibration, tuning, matching, pulse calibration, fixed receiver gain, and number of scans, were fully automated.

Spectral preprocessing was carried out in TopSpin. Manual phase correction was performed using the .ph command, followed by Fourier transformation (efp) and baseline correction (abs) to address baseline drift and ensure accurate peak quantification. ^1^H-NMR spectra were acquired at 300 K operating at a proton frequency of 600.20 MHz using an Avance III−600 Bruker spectrometer. One-dimensional ^1^H pulse experiments were carried out with a total of 32 scans using the nuclear Overhauser effect spectroscopy (NOESY)-presaturation sequence to suppress the residual water peak at around 4.7 ppm. This 1D NOESY-based sequence includes presaturation during the relaxation delay (D1 = 5 s) for efficient water suppression. The acquisition time (AQ) was 3.42 s, and the total experiment duration was 4 min and 42 s per sample, providing an adequate signal-to-noise ratio and resolution for metabolomic profiling.

**Figure 1:**
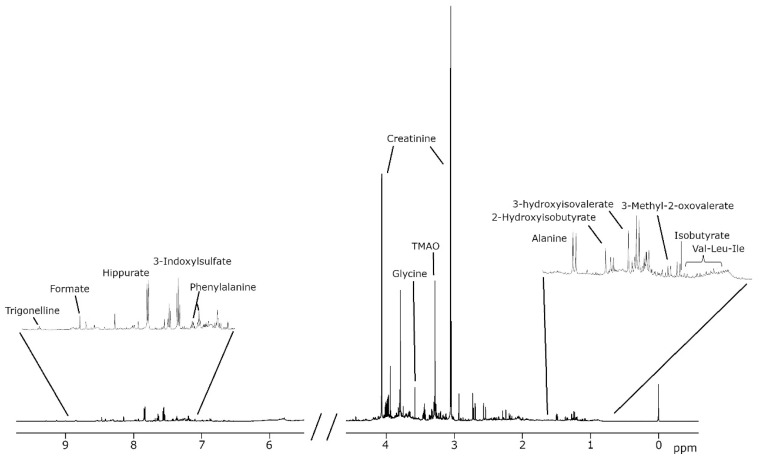
Representative Nuclear Magnetic Resonance (NMR) spectra highlighting the most relevant peaks corresponding to the urinary metabolites identified in the analysis.

For spectral referencing, all spectra were aligned to the sodium 3-(trimethylsilyl)propionate (TSP) signal at 0.00 ppm. After this initial global alignment, spectra were divided into specific regions to allow for localized, fine-tuned alignment. This second alignment step corrects small peak shifts, typically resulting from pH variations. Once aligned, spectral deconvolution algorithms were used to reconstruct the raw signal using predefined analytical peak shapes (e.g., singlets, doublets, triplets) based on known metabolite patterns. These functions were iteratively fitted to the spectra to minimize residual error, enabling precise peak boundary definition and accurate metabolite quantification. The NMR area, chemical shift (ppm), and signal assignment associated with the concentration of 34 metabolites (Table 1) were obtained after the spectral analysis (Figure 1) using an in-house lineshape fitting algorithm developed to deconvolute the pre-processed NMR spectra, based on Lorentzian and Gaussian functions to minimize the fitting error, as previously described [34]. The NMR areas were transformed into concentration units by using specific conversion factors depending on the proton numbers of the molecular structure generating the signal. Finally, the metabolite concentrations were normalized by using Probabilistic Quotient Normalization (PQN) to avoid urine dilution effects [35].

### 2.3. Statistical Analysis

For the descriptive analysis, biochemical variables were summarized using the median and interquartile range (IQR) to account for data distribution. Normality of the variables was assessed using the Shapiro–Wilk test [36]. Given that several variables did not follow a normal distribution, the Wilcoxon–Mann–Whitney rank-sum test was used for unadjusted comparisons of urinary metabolite concentrations and dietary intake between groups defined by sex and EAT-Lancet adherence [37]. Additionally, to further explore the relationship between sex, dietary adherence, and biomarker levels while adjusting for confounding variables (age, BMI, and total energy intake), analysis of covariance (ANCOVA) models was applied [38]. Interaction terms were included to test for effect modification by sex. To explore associations between dietary categories and metabolite concentrations, pairwise Spearman correlation analyses were performed [39].

For the inferential analysis, linear regression models were employed to evaluate the relationship between metabolite levels and food group dietary intake, adjusting for potential confounders such as age (years), BMI (kg/m^2^), and total energy intake (kcal/d) [40]. The association between metabolite levels and food group dietary intake from the FFQ was analyzed separately for men and women. Scatter plots were generated to illustrate the predicted versus observed values for each sex, with fitted regression lines providing a representation of the trends. Mean concentrations of metabolites were also compared across sex and different levels of food group dietary intake to assess potential dietary influences on metabolite levels. These strategies are widely recognized and produce plausible outcomes.

Given the exploratory nature of the study, the analyses aimed to identify potential associations to confirm predefined hypotheses. As such, corrections for multiple testing (e.g., Bonferroni or Sidak) were not applied in the correlation or regression models. This approach is consistent with methodological recommendations for hypothesis-generating studies in metabolomics, which caution against overcorrection due to the increased risk of type II errors and the loss of potentially relevant findings [41].

All analyses were performed using Stata version 16.0 (StataCorp, College Station, TX, USA). All *p*-values are two-sided, with statistical significance set at *p* < 0.05.

## 3. Results

Table 2 summarizes the EAT-Lancet score components for the 138 study participants, stratified into high (>P50) and low (<P50) adherence groups. Women represented the majority in both groups, especially among high adherents (79.4%). Men with low adherence consumed fewer grains (120.6 vs. 162 g/day; *p* = 0.027) and more red meat (115.2 vs. 161.9 g/day; *p* = 0.001) than women. Conversely, women with low adherence had lower fruit intake than men (210.7 vs. 345.4 g/day; *p* = 0.003) and consumed less fish (*p* = 0.037). Overall, the EAT-Lancet score effectively distinguished fruit, dairy, poultry, and nut intake between adherence groups, with nut consumption notably higher—83% in women and 108% in men—in the high adherence group.

Metabolomic analysis revealed sex-specific differences in gut microbiota-related metabolites according to adherence level (Table 3). In women, urinary hippuric acid levels were similar across groups (3.934 vs. 3.846 mmol/L), whereas in men, they differed significantly (3.367 vs. 2.871 mmol/L), suggesting hippuric acid may serve as a dietary biomarker linked to the EAT-Lancet index in men. Full NMR metabolite data are available in Table S1.

**Figure 2:**
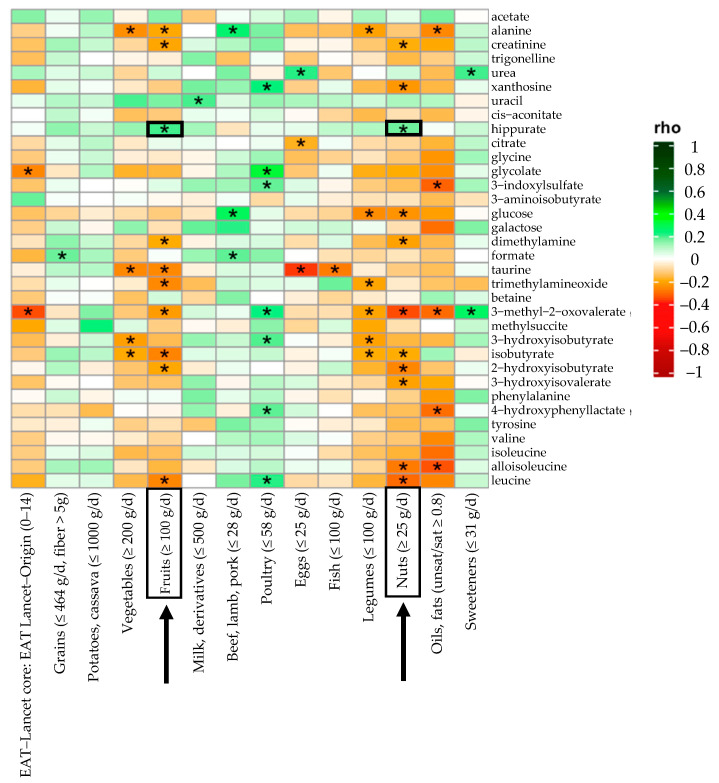
Spearman correlations between the 34 metabolites analyzed by Nuclear Magnetic Resonance (NMR) and the 14 categories of the EAT-Lancet score analyzed for all the volunteers of the Dietary Deal project (n = 138). Fruit and nut consumption is associated with hippuric acid concentration, which is indicated in the figure with arrows and boxes to facilitate identification. * *p* < 0.05.

Figure 2 shows a Spearman correlation heatmap illustrating associations between identified urinary metabolites and the 14 food groups of the EAT-Lancet score across all study participants. Heatmaps stratified by adherence level are available in Supplementary Figure S1. Positive correlations (in green) indicate higher metabolite levels with greater intake of specific food groups, while negative correlations (in orange-red) reflect inverse associations. Among all food categories, poultry showed the highest number of positive correlations (n = 7), mainly with amino acid-related metabolites. Red meat (beef, lamb, and pork) was positively linked to glucose, alanine, and formate. Other animal-based foods were associated with urea (eggs) and uracil (milk and dairy).

**Figure 3:**
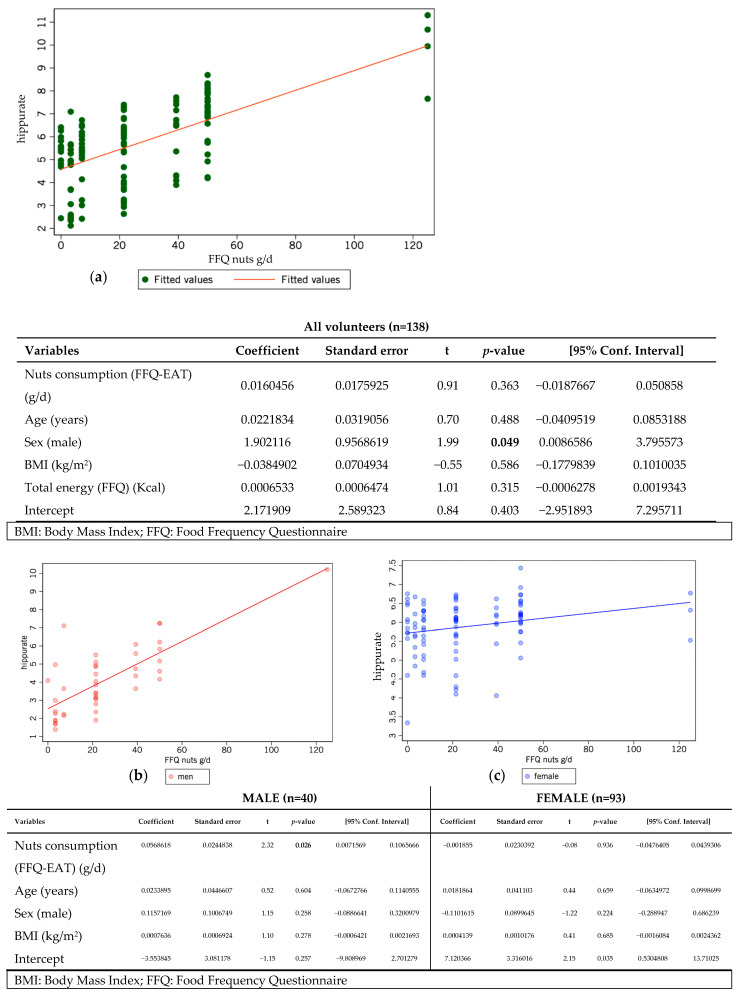
Linear regression analysis between hippuric acid (mmol/L) and nuts consumption (g/d) derived from the Food Frequency Questionnaire (FFQ) among participants in the Dietary Deal project. Portion size was described mostly between 0 and 70 g/d, with only a few participants with consumption higher than 100 g/d (**a**). More specific analysis was performed independently in men (**b**) and women (**c**). Extreme values (<P5 and >P95) were excluded, and the model was adjusted for age, sex, Body Mass Index (BMI), and total energy intake. The model explained 24% of the variability in hippurate levels. In men, higher intake of nuts (g/day) was positively associated with hippurate levels, independent of age, BMI, and total energy intake (β = 0.057, 95% CI: 0.007 to 0.107, *p* = 0.026). Conversely, in women, nut intake showed no significant association (β = –0.002, 95% CI: –0.048 to 0.044, *p* = 0.936), and the model had very limited explanatory power (R^2^ = 0.02).

Hippuric acid showed positive correlations with fruit and nut intake, standing out as the plant-based metabolite most associated with EAT-Lancet adherence, and for the first time, specifically linked to nut consumption. Conversely, the most negative correlations were found in plant-based categories, with ten and nine metabolites negatively associated with nut and fruit intake, respectively, reflecting distinct metabolic responses to these food groups. Taurine was the only metabolite inversely correlated with animal-derived foods (eggs, fish). Higher overall adherence to the EAT-Lancet score was also associated with lower levels of 3-methyl-2-oxovalerate and glycolate. Figure 3 presents results from a linear regression model examining the relationship between hippuric acid and nut intake, estimated via FFQ and stratified by sex. The model excluded extreme values as outliers (<P5–>P95) and was adjusted for age, sex, BMI, and total energy intake.

A positive slope was observed across all participants (Figure 3a), indicating a more consistent trend between nut consumption and hippuric acid levels, particularly for intake ranges between 0 and 40 g/day. Although data dispersion was greater at low consumption levels, hippuric acid still tended to increase with intake. When sex was considered (Figure 3b,c), both men and women showed a positive association, with most data points again in the lower to moderate intake range. However, the slope for women appeared steeper, suggesting a more detectable association. Although overall hippuric acid concentrations were higher in women, stratified analyses showed a more pronounced effect of nut intake on hippuric acid levels in men (*p* = 0.026), while no association was observed in women (*p* = 0.936).

**Figure 4:**
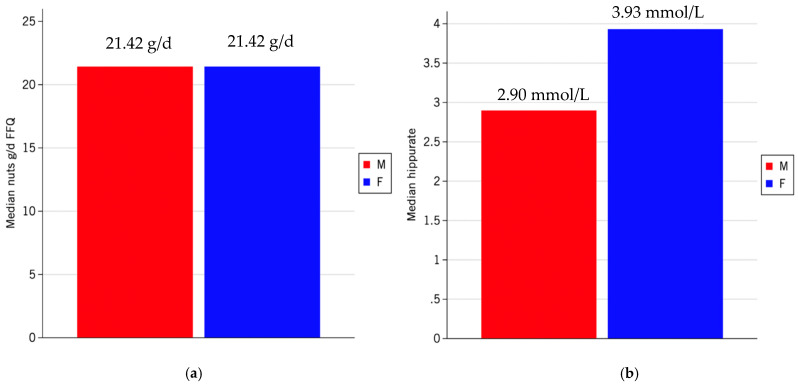
Average nut intake derived from the Food Frequency Questionnaire (FFQ) (g/d) expressed by median (P50) in males (M) and females (F) (**a**). Highlighting hippuric acid concentration, with the same nut consumption, males and females described different concentrations (**b**).

The bar graphs in Figure 4 show the average concentration (P50) of nut consumption between men and women with high adherence to the EAT-Lancet index. Nut intake is similar between sexes (Figure 4a) with no significant differences (*p* = 0.938). However, the hippuric acid concentrations are significantly lower in men compared to women (*p* = 0.020) (Figure 4b).

## 4. Discussion

This study explores the potential of urinary hippuric acid as a sex-dependent biomarker for fruit and nut intake, assessed in a plant-based diet adherence through the EAT-Lancet score. Although hippuric acid has been widely associated with the intake of fruits and vegetables due to the microbial metabolism of dietary polyphenols [21,42,43], the present analysis provides one of the first observations of a specific association with nut consumption. Our analysis expands on this knowledge by identifying, for the first time, a specific association with nut intake. Nuts such as walnuts and hazelnuts contain significant levels of ellagitannins and proanthocyanidins [44], which gut microbiota can convert into urolithins and benzoic acid derivatives—including hippuric acid—through colonic fermentation [45]. This metabolic pathway may explain the observed positive relationship between nut intake and hippuric acid concentration in urine samples.

In addition to this novel association, the study addresses sex-specific differences in hippuric acid excretion. Although women generally showed higher hippuric acid levels, a statistically significant association between nut consumption and hippuric acid was only detected in men (*p* = 0.026), not in women (*p* = 0.936). This suggests a more linear or detectable metabolic response to nut-derived polyphenols in men, rather than a stronger physiological effect. Differences in microbiota composition [46,47], colonic transit time and polyphenol absorption efficiency [48], and hepatic conjugation [21] could contribute to this disparity associated with sex. Hormonal influences may also play a role; for instance, estrogen modulates gut microbiota composition and intestinal permeability [43], while sex-specific differences in glycine availability and mitochondrial function may affect benzoic acid conjugation [47,49]. In fact, available research suggests that sex may influence urinary hippuric acid concentrations [50].

On the other hand, the exploratory nature of this study warrants cautious interpretation. The goal was to identify emerging associations that could inform future hypothesis-driven research. As supported by Menyhart et al. [42], multiple testing corrections are not always required in exploratory metabolomics, as they may increase type II errors and obscure meaningful associations.

However, our findings reinforce the relevance of hippuric acid as a marker not only for fruit and vegetable intake, but also for nut consumption. The integration of metabolomics with dietary indices, such as the EAT-Lancet score, revealed distinct correlations between metabolite concentrations and food categories. Plant-based groups like “Fruits”, “Vegetables”, and “Legumes” showed different associative patterns than animal-based categories such as “Meat”, “Dairy”, and “Fish”. For instance, alanine and glucose were positively associated with red meat intake, and urea was linked to egg consumption, reflecting dietary behaviors through metabolomic profiling. In contrast, taurine showed negative associations with plant-based foods, further emphasizing metabolic distinctions between dietary patterns [51,52].

Sex differences in metabolite levels were evident. While women exhibited higher absolute concentrations of hippuric acid, the association between nut intake and hippuric acid levels was only statistically detectable in men. Prior studies suggest that women may exhibit different metabolic responses due to differences in hormone profiles, body composition, and enzymatic activity [43,53,54]. These results underscore the need for sex-specific validation of dietary biomarkers, which may enhance the precision of personalized nutrition strategies.

A key strength of this study is the use of NMR-based metabolomics, which offers robust and reproducible analysis of urinary metabolites. Compared to mass spectrometry (MS), NMR requires minimal sample preparation, avoids issues such as ion suppression, and provides absolute quantification [34,55]. It is particularly well-suited for detecting structurally complex compounds like hippuric acid. Furthermore, NMR enables a holistic assessment of metabolic networks, allowing us to capture sex-related differences in polyphenol metabolism [47]. While MS remains the preferred method for highly sensitive detection, NMR is advantageous for large-scale applications requiring consistency and throughput [56].

Despite these strengths, the study has limitations. The observational design and reliance on FFQs may introduce self-report bias. Additionally, the relatively small sample size, appropriate for a pilot study, limits generalizability. Given the exploratory nature of this analysis and the sample size, multiple testing corrections (e.g., Bonferroni or Sidak) were not applied, as these could increase the risk of false negatives (type II errors) and hinder the detection of biologically relevant associations. Future studies should include larger, more diverse populations and consider integrating microbiota profiling to confirm the proposed mechanisms [57].

Finally, these results support the integration of metabolomics into personalized nutrition. As tools from genetics, microbiomics, and metabolomics advance, the ability to tailor dietary recommendations to individual profiles improves. This study contributes to this field by identifying hippuric acid as a promising candidate biomarker of nut intake, particularly in men, highlighting the importance of accounting for sex in dietary biomarker research and nutritional intervention design.

## Figures and Tables

**Table 1:** List of 34 metabolites analyzed by Proton Nuclear Magnetic Resonance (^1^H-NMR), including their chemical shifts (ppm) and signal assignment, categorized into six putative groups based on their origins (macronutrient metabolism—fats, proteins, carbohydrates, gut microbiota metabolism, nitrogen metabolism, and nucleotide metabolism).

Metabolism	Putative Associated Group	Compound Analyzed	Chemical Shift (ppm)	Signal
Amino acids or derived compounds	Aliphatic amino acids and derived compounds	Alanine	1.5	D
		Alloisoleucine	0.9	D
		Glycine	3.6	S
		Isoleucine	1.0	D
		Leucine	1.0	T
		Valine	1.0	D
	Aromatic amino acids and derived compounds	4-Hydroxyphenyllactate	6.9	M
		Phenylalanine	7.4	M
		Tyrosine	6.9	M
	Sulfonic acid	Taurine	3.3	T
Energy and carbohydrate metabolism	Krebs cycle	cis-Aconitate	5.7	M
		Citrate	2.7	M
		Methylsuccinate	1.1	D
	Carbohydrate metabolism	Galactose	5.3	D
		Glucose	5.2	D
		Glycolate	4.0	S
Gut microbiota metabolism	Carboxylic acids	Acetate	1.9	S
		Formate	8.5	S
	Metabolites derived from nitrogenous compounds	Dimethylamine	2.7	S
		Trimethylamine N-oxide (TMAO)	3.3	S
	Phenolic compounds related to dietary polyphenols	Hippurate	7.8	M
	Sulfur-containing compounds derived from amino acids	3-Indoxylsulfate	7.7	M
Nitrogenous compounds	Nitrogen-related metabolites	Creatinine	4.1	S
		Urea	5.8	S
	Methylation-related metabolites	Betaine	3.3	S
		Trigonelline	9.1	S
Nucleotide metabolism	Precursors or intermediates of nucleotides	Uracil	5.8	D
		Xanthosine	5.9	D
	Degradation products of nucleotides	3-Aminoisobutyrate	1.2	D
Fatty acid metabolism	Intermediates in branched-chain fatty acid oxidation	3-Methyl-2-oxovalerate	1.1	D
		3-Hydroxyisovalerate	1.3	S
	Metabolites from beta-oxidation and alternative pathways	3-Hydroxyisobutyrate	1.1	D
	Short-chain fatty acids (SCFAs)	Isobutyrate	1.1	D
		2-Hydroxyisobutyrate	1.4	S

S: Singlet, an isolated proton with no neighboring protons; D: Doublet, a proton with one neighboring proton; T: Triplet, a proton with two equivalent neighboring protons; M: Multiplet, a proton coupled to multiple non-equivalent protons or involved in more complex systems.

**Table 2:** Description of the 14 components of the EAT-Lancet score based on low or high adherence according to the median (P_50_), differentiated by sex.

	EAT Lancet Score (n = 138)	EAT-Lancet Score < P_50_ (n = 75)			EAT-Lancet Score ≥ P_50_ (n = 63)				
	Total (n = 138)	Male (n = 29)	Female (n = 46)		Male (n = 13)	Female (n = 50)			
EAT Lancet food group (g/d)	P_50_ (IQR)	P_50_ (IQR)	P_50_ (IQR)	*p*	P_50_ (IQR)	P_50_ (IQR)	*p*	p-EAT	p-Sex
EAT-Lancet Score (0–14p)	8 (7–9)	8 (7–8)	8 (7–8)	0.732	9 (9–10)	9 (9–10)	0.826	<0.001	0.316
Grains	120.6 (79.3–169.1)	162 (103.2–191.4)	100.8 (60–173.3)	0.027	126.2 (103.3–171.3)	107.6 (77–161.4)	0.344	0.504	0.010
Potatoes	31.4 (20–74.3)	42.8 (21.4–85.7)	31.4 (20–64.3)	0.151	53.6 (10–74.3)	31.4 (20–64.3)	0.737	0.385	0.148
Vegetables	473.1 (364.2–808.2)	416.6 (307.6–594.5)	504.6 (349.9–754.7)	0.262	593.3 (399.9–776)	469 (378.5–828.5)	0.985	0.168	0.282
Fruits	345.4 (196.8–499.2)	210.7 (124.6–286.8)	358.5 (222.7–474.7)	0.003	353.4 (267.8–542.3)	372.8 (207.1–613.5)	0.591	0.012	0.005
Milk and derivatives	347.1 (226–558.6)	342.2 (240.1–607.1)	503.3 (276.6–564.3)	0.960	285.5 (202.8–365.1)	299 (142.6–517.6)	0.725	0.010	0.790
Beef, lamb, pork	115.2 (77.3–160)	161.9 (113.5–189.3)	104.2 (71.4–149.9)	0.001	125 (84.7–178.8)	98.8 (51.9–142.8)	0.335	0.096	0.001
Poultry	64.3 (31.4–74.3)	64.3 (64.3–74.3)	64.3 (64.3–74.3)	0.498	64.3 (31.4–74.3)	31.4 (21.4–64.3)	0.144	<0.001	0.045
Eggs	25.7 (25.7–47.1)	25.7 (25.7–47.1)	25.7 (25.7–47.1)	0.621	25.7 (25.7–47.1)	25.7 (25.7–47.1)	0.476	0.676	0.375
Fish	81.6 (56.2–117)	78.7 (45.1–109.8)	94.9 (62–137.8)	0.037	89.5 (64.3–103)	77.3 (56.8–98.9)	0.239	0.139	0.349
Legumes	17.1 (12.6–29.7)	16.8 (16–27.4)	16.6 (12–25.7)	0.600	25.4 (16–46.8)	21.1 (12.6–29.7)	0.200	0.110	0.364
Nuts	21.4 (7.1–39.3)	21.4 (3.3–21.4)	21.4 (7.1–21.4)	0.828	44.6 (21.4–50)	39.3 (7.1–50)	0.484	<0.001	0.934
Oils, fats (unsat/sat ratio)	18 (5.8–37.5)	11.7 (4.7–18.5)	22.6 (5.8–37.5)	0.331	14.5 (14–35)	24 (5–40.8)	0.919	0.941	0.456
Sweeteners	1.4 (0–10)	8.6 (0.3–25)	1.3 (0–10.7)	0.129	0 (0–7.9)	1.4 (0–10)	0.101	0.064	0.657

IQR: Interquartile range. Grains: score of 1 for consumption ≤ 464 g/day and fiber > 5 g; potatoes and cassava: score of 1 for consumption ≤ 100 g/day; vegetables: score of 1 for consumption ≥ 200 g/day; fruits: score of 1 for consumption ≥ 100 g/day; milk and dairy products: score of 1 for consumption ≤ 500 g/day; beef, lamb, and pork: score of 1 for consumption ≤ 28 g/day; poultry: score of 1 for consumption ≤ 58 g/day; eggs: score of 1 for consumption ≤ 25 g/day; fish: score of 1 for consumption ≤ 100 g/day; legumes: score of 1 for consumption ≤ 100 g/day; nuts and seeds: score of 1 for consumption ≥ 25 g/day; oils and fats: score of 1 if the unsaturated-to-saturated fat ratio is ≥0.8; sweeteners: score of 1 for consumption ≤ 31 g/day.

**Table 3:** Concentration of gut microbiota metabolism (mmol/L) in urine samples from volunteers in the Dietary Deal study (n = 138) analyzed by Proton Nuclear Magnetic Resonance (^1^H-NMR) based on low or high adherence according to the median (P_50_) using the EAT-Lancet Score.

			EAT-Lancet < Median (n = 75)			EAT-Lancet ≥ Median (n = 63)				
		Total (n = 138)	Male (n = 29)	Female (n = 46)		Male (n = 13)	Female (n = 50)			
Metabolite (mmol/L)	Missing/Total	P50 (IR)	P50 (IQR)	P50 (IQR)	*p*	P50 (IQR)	P50 (IQR)	*p*	p-EAT	p-Sex
3-indoxylsulfate	14/138	0.347 (0.2–0.55)	0.349 (0.178–0.609)	0.38 (0.26–0.621)	0.659	0.342 (0.133–0.46)	0.292 (0.199–0.454)	0.876	0.074	0.783
Acetate	28/138	0.041 (0.023–0.066)	0.039 (0.015–0.058)	0.035 (0.024–0.068)	0.210	0.058 (0.032–0.127)	0.042 (0.024–0.1)	0.383	0.292	0.688
Dimethylamine	1/138	0.542 (0.371–0.776)	0.617 (0.443–0.896)	0.552 (0.374–0.778)	0.461	0.522 (0.354–0.736)	0.493 (0.361–0.732)	0.926	0.249	0.475
Formate	8/138	0.249 (0.169–0.353)	0.214 (0.174–0.401)	0.289 (0.202–0.419)	0.417	0.224 (0.182–0.297)	0.196 (0.147–0.295)	0.474	0.020	0.904
Hippurate	5/138	3.589 (2.252–6.8)	3.367 (1.611–5.121)	3.934 (2.784–7.294)	0.045	2.871 (2.372–4.236)	3.846 (2.105–7.668)	0.218	0.909	0.020
Trimethylamine-N-oxide	35/138	0.681 (0.417–1.776)	1.032 (0.438–1.436)	0.621 (0.417–1.828)	0.653	0.539 (0.279–0.873)	0.612 (0.393–1.486)	0.344	0.337	0.838

IQR: Interquartile range.

## Data Availability

The raw data supporting the conclusions of this article will be made available by the authors on request.

## Supplementary Materials

The following supporting information can be downloaded at: https://www.mdpi.com/article/10.3390/metabo15060348/s1. Figure S1: Spearman correlations between high (A) and low (B) adherence to EAT-Lancet score according to median (P50) of the Dietary Deal project (n = 138); Table S1: Concentration of metabolites (mmol/L) different from gut microbiota in urine samples from volunteers in the Dietary Deal study (n = 138) analyzed by Nuclear Magnetic Resonance (^1^H-NMR) based on low or high adherence according to the median (P50) using the EAT-Lancet Score.
