# Supplementary material for: Urinary Hippuric Acid as a Sex-Dependent Biomarker for Fruit and Nut Intake Raised from the EAT-Lancet Index and Nuclear Magnetic Resonance Analysis
Source: Metabolites. 2025 May 23;15(6):348. doi: 10.3390/metabo15060348 (PMC12194962; doi:10.3390/metabo15060348)

Table S1. Concentration of metabolites (mmol/L) different from gut microbiota in urine samples from volunteers in the Dietary Deal study (n=138) analyzed by Nuclear Magnetic Resonance (1^{H}-NMR) based on low or high adherence according to the median (P50) using the EAT-Lancet Score.

			EAT-Lancet < median (n=75)			EAT-Lancet ≥ median (n=63)					
		Total (n=138)	Male (n=29)	Female (n=46)		Male (n=13)	Female (n=50)				
Metabolite (mmol/L)	missing/total	P50 (IR)	P50 (IR)	P50 (IR)	p	P50 (IR)	P50 (IR)	p	p-EAT	p-sex	p-int sex#EAT
**Amino acids and derived compounds**											
**4-hydroxyphenyllactate**	5/138	0.316 (0.202-0.46)	0.306 (0.196-0.47)	0.334 (0.215-0.5)	0.720	0.248 (0.164-0.483)	0.291 (0.206-0.411)	0.580	0.258	0.649	0.944
**Alanine**	4/138	0.389 (0.28-0.655)	0.537 (0.324-0.86)	0.358 (0.273-0.639)	0.048	0.398 (0.228-0.734)	0.339 (0.263-0.503)	0.679	0.144	0.042	0.858
**Alloisoleucine**	35/138	0.009 (0.005-0.017)	0.009 (0.005-0.022)	0.009 (0.006-0.017)	0.788	0.014 (0.006-0.026)	0.006 (0.004-0.011)	0.082	0.253	0.162	0.529
**Glycine**	3/138	1.578 (0.995-2.854)	1.442 (1.028-2.884)	1.948 (1.104-3.351)	0.469	1.131 (0.737-2.028)	1.578 (0.943-2.538)	0.541	0.175	0.509	0.280
**Isoleucine**	43/138	0.032 (0.019-0.055)	0.03 (0.021-0.047)	0.037 (0.024-0.061)	0.334	0.027 (0.017-0.035)	0.025 (0.016-0.055)	0.898	0.149	0.442	0.823
**Leucine**	22/138	0.046 (0.027-0.072)	0.069 (0.042-0.099)	0.048 (0.028-0.062)	0.060	0.052 (0.023-0.082)	0.035 (0.022-0.055)	0.176	0.018	0.012	0.867
**Phenylalanine**	10/138	1.522 (1.045-2.29)	1.46 (0.817-2.38)	1.677 (1.137-2.571)	0.285	1.246 (0.747-1.97)	1.615 (1.154-1.876)	0.188	0.494	0.103	0.244
**Taurine**	66/138	1.93 (0.925-3.536)	2.234 (0.956-3.817)	2.009 (0.893-3.379)	0.480	2.522 (0.853-4.226)	1.778 (1.046-2.451)	0.538	0.652	0.348	0.920
**Tyrosine**	21/138	0.208 (0.166-0.282)	0.283 (0.178-0.464)	0.203 (0.162-0.256)	0.002	0.24 (0.112-0.358)	0.179 (0.165-0.232)	0.728	0.073	0.002	0.141
**Valine**	7/138	0.063 (0.042-0.094)	0.067 (0.044-0.097)	0.069 (0.043-0.097)	0.910	0.062 (0.035-0.124)	0.057 (0.035-0.079)	0.470	0.191	0.433	0.312
**Energy and carbohydrate metabolism**											
**Cis-Aconitate**	34/138	0.275 (0.189-0.387)	0.311 (0.206-0.375)	0.275 (0.189-0.401)	1.000	0.272 (0.186-0.419)	0.268 (0.186-0.374)	0.578	0.568	0.678	0.794
**Citrate**	0/138	3.327 (2.164-5.106)	2.941 (2.227-4.399)	3.664 (2.76-6.353)	0.035	3.087 (1.372-4.274)	3.109 (1.887-4.98)	0.553	0.178	0.075	0.253
**Galactose**	75/138	0.212 (0.143-0.31)	0.289 (0.188-0.362)	0.178 (0.143-0.292)	0.292	0.304 (0.264-0.435)	0.181 (0.132-0.241)	0.030	0.533	0.029	0.053
**Glucose**	58/138	1.598 (1.335-1.916)	1.598 (1.421-1.881)	1.723 (1.438-1.967)	0.349	1.622 (1.003-2.743)	1.377 (1.187-1.808)	0.602	0.082	0.918	0.073
**Glycolate**	53/138	1.131 (0.703-1.762)	1.385 (0.926-1.819)	1.255 (0.703-2.093)	0.872	1.217 (1.063-2.226)	0.859 (0.508-1.243)	0.032	0.054	0.093	0.394

**Nitrogenous compounds**											
**Betaine**	94/138	0.09 (0.049-0.147)	0.1 (0.048-0.164)	0.1 (0.072-0.136)	0.854	0.109 (0.058-0.235)	0.067 (0.036-0.091)	0.325	0.226	0.328	0.579
**Creatinine**	0/138	17.67 (11.814-24.693)	23.829 (15.424-29.658)	18.034 (10.292-24.487)	0.034	15.803 (10.23-29.259)	15.325 (11.614-20.273)	0.882	0.032	0.031	0.559
**Trigonelline**	5/138	0.3 (0.193-0.479)	0.224 (0.161-0.378)	0.373 (0.258-0.582)	0.009	0.213 (0.151-0.273)	0.303 (0.176-0.479)	0.075	0.182	0.002	0.863
**Urea**	2/138	2.913 (2.42-3.384)	2.927 (2.424-3.242)	2.72 (2.252-3.271)	0.502	2.854 (2.412-4.298)	3.142 (2.624-3.472)	0.458	0.063	0.800	0.537
**Nucleotide metabolism**											
**Uracil**	39/138	0.046 (0.018-0.092)	0.021 (0.016-0.057)	0.062 (0.023-0.127)	0.038	0.059 (0.018-0.15)	0.045 (0.023-0.064)	0.355	0.933	0.262	0.010
**Xanthosine**	23/138	0.224 (0.17-0.298)	0.219 (0.188-0.353)	0.259 (0.158-0.338)	0.774	0.212 (0.142-0.366)	0.202 (0.17-0.271)	0.990	0.094	0.508	0.804
**Fatty acid metabolism**											
**2-hydroxyisobutyrate**	7/138	0.068 (0.046-0.094)	0.079 (0.046-0.107)	0.065 (0.044-0.112)	0.483	0.062 (0.038-0.101)	0.065 (0.049-0.085)	0.921	0.309	0.376	0.646
**3-aminoisobutyrate**	20/138	0.155 (0.074-0.301)	0.108 (0.069-0.189)	0.127 (0.063-0.284)	0.348	0.282 (0.215-0.493)	0.183 (0.077-0.372)	0.093	0.027	0.808	0.226
**3-hydroxyisobutyrate**	6/138	0.155 (0.101-0.247)	0.176 (0.124-0.247)	0.173 (0.113-0.258)	0.909	0.139 (0.085-0.302)	0.138 (0.099-0.195)	0.762	0.092	0.563	0.367
**3-hydroxyisovalerate**	5/138	0.532 (0.342-0.806)	0.553 (0.343-0.886)	0.586 (0.355-0.841)	0.789	0.651 (0.349-0.803)	0.412 (0.301-0.731)	0.271	0.251	0.549	0.351
**3-methyl-2-oxovalerate**	29/138	0.218 (0.152-0.295)	0.237 (0.198-0.369)	0.249 (0.199-0.321)	0.894	0.162 (0.128-0.262)	0.18 (0.148-0.23)	0.857	<0.001	0.626	0.558
**isobutyrate**	14/138	0.011 (0.006-0.018)	0.014 (0.007-0.021)	0.012 (0.007-0.016)	0.299	0.008 (0.004-0.014)	0.009 (0.004-0.016)	0.918	0.059	0.385	0.389
**methylsuccite**	71/138	0.025 (0.013-0.052)	0.023 (0.012-0.042)	0.033 (0.019-0.063)	0.128	0.009 (0.008-0.041)	0.019 (0.013-0.036)	0.417	0.052	0.212	0.808

Figure S1. Spearman correlations between high (A) and low (B) adherence to EAT-Lancet score according to median (P50) of the Dietary Deal project (n=138).

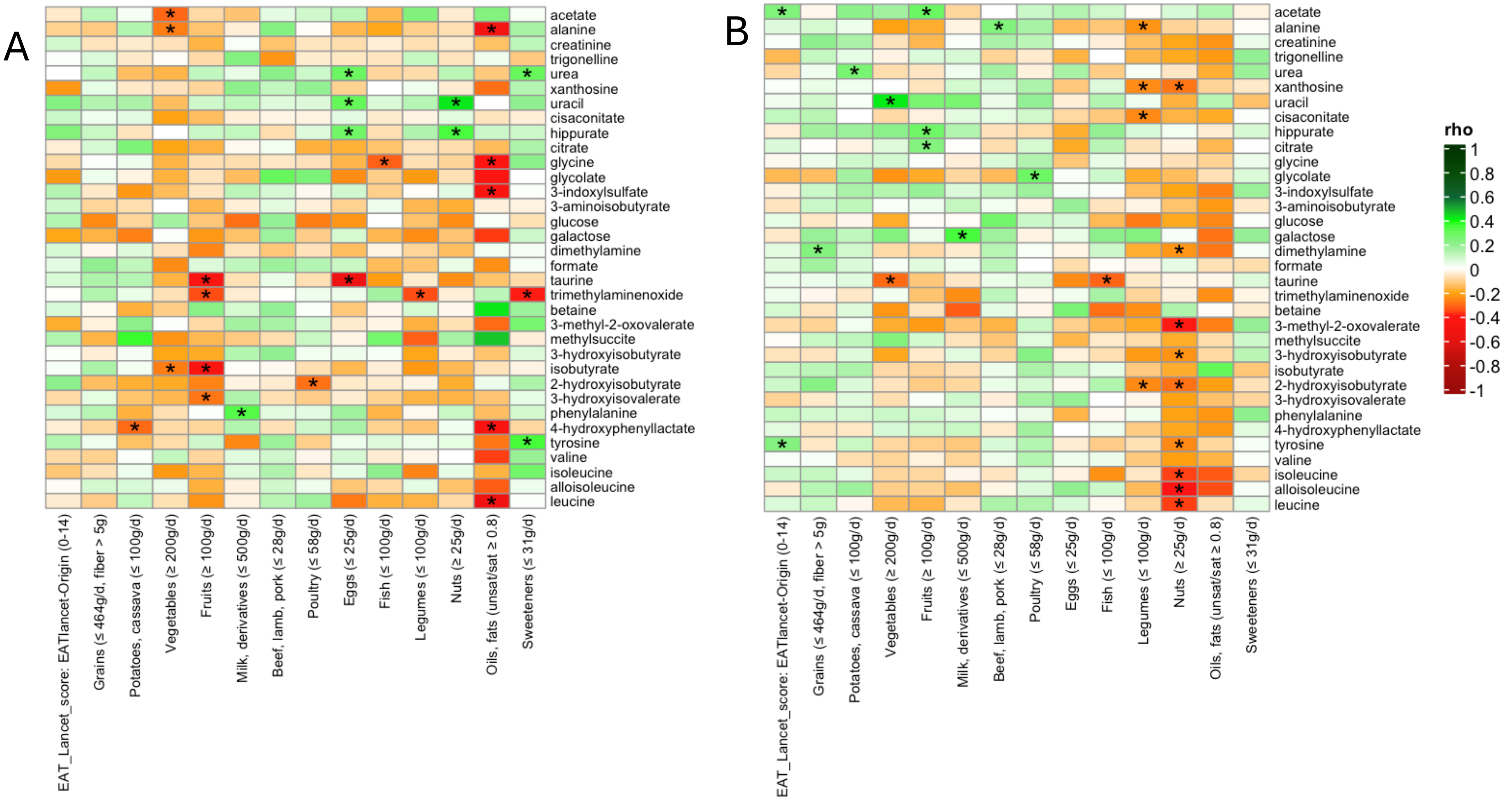

Supplement: Supplementary file 1 [file metabolites-15-00348-s001.zip › metabolites-3584603-supplementary.pdf]
